# Factors Associated With Scoliosis in Schoolchildren: a Cross-Sectional Population-Based Study

**DOI:** 10.2188/jea.JE20140061

**Published:** 2015-03-05

**Authors:** Marina Pegoraro Baroni, Geronimo José Bouzas Sanchis, Sanderson José Costa de Assis, Rafael Gomes dos Santos, Silvana Alves Pereira, Klayton Galante Sousa, Johnnatas Mikael Lopes

**Affiliations:** 1 Physiotherapy, Graduate Program at the Mid-West State University, Paraná, Brazil; 2 Graduate Physiotherapist from the Federal University of Rio Grande do Norte, Rio Grande do Norte, Brazil; 3 Multidisciplinary Maternal and Child Residency at Hospital Ana Bezerra, Federal University of Rio Grande do Norte, Rio Grande do Norte, Brazil; 4 Physiotherapy; Doctoral student at the Brasília University, Federal District, Brazil; 5 Physiotherapy, Graduate Program at the Federal University of Rio Grande do Norte, Rio Grande do Norte, Brazil

**Keywords:** scoliosis, children, risk factors

## Abstract

**Background:**

The present study aimed to investigate the prevalence of scoliosis and to analyze the factors associated with scoliosis in schoolchildren aged between 7 and 17 years.

**Methods:**

This is a cross-sectional and quantitative study with stratified random selection of public school students in the city of Santa Cruz, Brazil. The presence of scoliosis was examined, as well as the flexibility of the posterior muscle chain, socioeconomic characteristics, anthropometry, lifestyle habits, sexual maturation, and ergonomics of school furniture. In order to identify factors associated with scoliosis, the variables were divided in biological, socioeconomic, lifestyle, and ergonomic factors, and crude and adjusted prevalence ratios (PRs) were estimated by means of Poisson regression analysis.

**Results:**

Two hundred and twelve pupils participated in this study (mean age 11.61 years, 58% female). The prevalence of scoliosis was 58.1% (*n* = 123) and associated with female sex (PR 2.54; 95% CI, 1.33–4.86) and age between 13 and 15 years (PR 5.35; 95% CI, 2.17–13.21). Sleeping in a hammock was inversely associated with scoliosis (PR 0.44; 95% CI, 0.23–0.81).

**Conclusions:**

Scoliosis seems to be positively associated with female sex and age between 13 and 15 years, whereas the habit of sleeping in a hammock is negatively associated with the onset of scoliosis.

## INTRODUCTION

Posture is the relationship between muscles, bones, and joints, and posture adapts to internal and external stimuli. Good posture comes from the balance between these structures, reducing energy consumption and discomfort.^1^^,^^2^

Genetics are known to play an important role in the etiology of scoliosis. As with other chronic non-communicable diseases, scoliosis shows an epigenetic aspect that is characterized by interaction between environmental factors and the genome in modulation of phenotypic differences. The epigenetic effects mainly occur through DNA methylation in the first decade of life, due to internal and external environment exposure.^3^

There are several risk factors that promote changes in posture, including heredity, inadequate postural habits, low physical activity, overweight and obesity, and other factors.^2^ In addition, the school environment may be detrimental to good postures due to the presence of risk factors such as: inadequate school furniture, sitting for long periods of time, asymmetric straps on school backpacks and/or backpacks overloaded with school supplies, the use of inappropriate footwear, and other factors.^2^^,^^4^^,^^5^ In addition to these risk factors, the socioeconomic level of the family seems to be related with the presence of scoliosis.^6^ Although we cannot assert that public school students are all needy, it has been observed that most public school students in Brazil originated in disadvantaged economic classes, and that, from 6 to 7 years of age, height reflects socioeconomic and environmental factors in which the child lives.^6^ Besides the lack of balanced nutrition, regular use of appropriate footwear and other modulating factors affect phenotype.^7^

These risk behaviors related to posture are associated with periods of growth spurt, which contribute to the emergence of postural changes in childhood and adolescence, particularly scoliosis.^8^^–^^11^ According to Fornazari and Pereira,^11^ the prevalence of scoliosis in school-aged children is around 22% in the Brazilian population. Postural problems tend to worsen in adolescence and adulthood, resulting in spinal diseases, which have become a public health issue.^12^ Assuming that epidemiological studies are the first step to developing disease prevention and health promotion programs,^13^ research is needed on the risk factors for postural problems to which school-aged children are exposed. Such research may justify the inclusion of postural assessment routines in schools, since this can provide an initial diagnosis of vertebral column disorders, as well as the factors related with its emergence.

The null hypotheses to be tested in this study are that biological and sociodemographic variables, lifestyle, and school furniture are factors that may not be associated with development of scoliosis in the school environment. Since epidemiological studies of schoolchildren from regions with low socioeconomic status in Brazil are scarce, we aimed to investigate the prevalence of scoliosis and to analyze the factors associated with scoliosis in school-aged children between 7 and 17 years old living in a city located in the Northeast region of Brazil.

## METHODOLOGY

This is an observational cross-sectional study with a quantitative approach conducted in the city of Santa Cruz, state of Rio Grande do Norte, Brazil. The study was approved by the Committee of Research Ethics Involving Humans at the Federal University of Rio Grande do Norte (UFRN; Approval No. 228/2011 and Protocol No. 052/11-P).

We included children aged between 7 and 17 years who were enrolled in elementary or secondary education at state or local urban schools in the morning or evening in 2011 and who agreed to participate in the research and for whom the terms of free and informed consent (TFIC) form was duly signed by the adult who was the minor’s guardian. Individuals with physical and/or mental disabilities or orthopedic, traumatology, and/or rheumatologic injuries that would hinder maintaining an orthostatic position were excluded from the study.

The sample size was determined assuming a prevalence of scoliosis of 26%^8^ using the StatCalc module of the Epiinfo 6.0 statistical package for a population of 5295 schoolchildren enrolled in 14 elementary and secondary public education schools in the municipality of Santa Cruz. This calculation resulted in a total of 192 schoolchildren to be assessed; this number was increased by 30% in order to account for probable losses, resulting in a sample of 250 schoolchildren. Sampling was probabilistic, stratified, and proportional to the total number of students in each school, and a random drawing using the numbered attendance list of each classroom was conducted.

We designed an assessment tool composed of an identification form (name, age and gender), suspected scoliosis assessment and assessment of the flexibility of the posterior muscle chain, and collection of socioeconomic characteristics, anthropometric measurements, life habits, sexual maturation, and ergonomic analysis. This data collection was performed by seven students of the Undergraduate Physiotherapy and Nursing Program at the School of Health Sciences in Trairi (FACISA), UFRN, who had been trained in advance.

The evaluators’ training and qualification consisted of four theoretical and practical meetings for familiarization with the assessment instruments. Subsequently, a pilot study of the assessment form was conducted among 12 volunteer students in order to analyze the inter-evaluator reliability. The assessment of interexaminer reliability was performed using the intraclass correlation coefficient (ICC), and variables were included in the assessment instrument when obtained ICC values were classified as having good or excellent reliability (ICC ≥ 0.80).

Teams of at least three college students were formed to collect data on days previously scheduled with the school board, according to the evaluators’ availability. Each team performed the schoolchildren’s assessment in its entirety, with male evaluators assessing boys and female evaluators assessing girls. All questionnaires used in the assessment were administered in a face-to-face interview.

We used the Adams forward bend test to assess suspected scoliosis, according to the methodology proposed by Santos.^14^ The evaluator was positioned behind the patient and asked the child to undertake a trunk flexion, inclining the head and allowing the arms to fall towards the ground. The evaluator observed the symmetry of the thoracic and lumbar spine in order to identify the presence of spinal deformity. Gibbosity was defined as the condition of over-curvature opposed to a contralateral flattening. The possible results for this test were: suspicion of scoliosis (gibbosity presence) or absence of scoliosis (gibbosity absence).^14^^,^^15^

Regarding posterior muscle chain flexibility, the tibiotarsal joint angle and the distance from hand to ground were checked. For the tibiotarsal joint angle analysis, children were told to incline their head, then their trunk, bringing hands toward the floor. A tibiotarsal joint angle greater than 90° indicates that the soleus muscle is retracted. Regarding the distance from hand to ground, the students were asked to place their right hands over their left, and were told to touch the ground with the fingertips. This variable was dichotomized as third finger touching the ground or not touching the ground.^14^

The family socioeconomic assessment was conducted according to the Brazilian criteria for economic classification, developed by the Brazilian Association of Research Companies (ABEP; *Associação Brasileira de Empresas de Pesquisa*).^16^ In cases of non-response, the head of the family was contacted by phone. Socioeconomic status was classified into two groups: one group with classes B1 and B2, and another with classes C, D, and E. Classes B1 and B2 refer to families with average monthly income between US $646.90 and US $1086.82. Classes C, D, and E refer to families with average monthly income lower than US $359.30.

The anthropometric variables assessed in the study were: body mass, height, and body mass index (BMI). Body mass was measured using a *Filizola* digital scale with 100-g precision. The procedures were performed with the barefoot students wearing light clothes.^17^ A wall-mounted stadiometer with foot plate and 1-mm precision was used to measure height. The students’ bodies were held in an orthostatic position (standing upright), barefoot and with their feet joined together, with their heads positioned horizontally after a maximal inspiration.^18^ BMI was calculated as the ratio between body mass and the square of height (kg/m^2^) and classified according to the reference values for age and gender suggested by the WHO.^19^

Life habits related to time watching television, the habit of sleeping in a hammock, and the hours of sleep were obtained through questions with open response options. Answers regarding the time watching television were dichotomized into ≤2 hours or >2 hours. The habit of sleeping in a hammock was categorized as “yes” for those who sleep in a hammock ≥4 times a week or “no” for those who sleep in a hammock <4 times a week. The hours of sleep were categorized as <8 hours, 8 to 10 hours, or >10 hours.

In order to assess the physical activity level, we applied the International Physical Activity Questionnaire (IPAQ) short format, which had been translated and validated for Brazil.^20^^,^^21^ Children were categorized into three levels of physical activity: individuals practicing moderate- or vigorous-intensity physical activities met the criteria of the active group, whereas other individuals were grouped in the low-active group, as recommended by the Canadian Paediatric Society.^22^

Maturational stage was assessed using the Tanner self-assessment of pubic hair, which shows a satisfactory agreement with medical assessment and was effective to determine the maturational stage in both males and females.^23^^,^^24^ The students were individually assessed in a private place inside their school, where drawings showing Tanner’s five stages of pubic hair development were posted, and students were told to identify the most similar figure to their current maturational stage. Students who had identified themselves as in stage 1 were considered prepubertal; those identified as in stages 2, 3, or 4 were considered pubertal; and those identified as in stage 5 were considered post-pubertal.^23^^,^^24^

Ergonomic assessment consisted of the school supplies weight (SSW) analysis, school supplies mode of transportation, and measuring the dimensions of the school’s desks and chairs. The SSW was measured using a *Filizola* digital portable scale with a 20-g precision. In order to check the adequacy of the SSW, the following classification was used: up to 5% of body mass for students less than 10 years old and up to 10% of body mass for students aged 10 years or older was considered appropriate; over 5% or 10% of body mass, respectively, was considered inappropriate.^9^

The school supplies mode of transportation was assessed through a questionnaire and classified as appropriate when transporting the material was performed using a two-strap backpack carried on both shoulders or using a trolley backpack; mode of transportation was considered inappropriate when school supplies were carried using any other means.

The analyzed dimensions of school desks were desk height (to ensure the desks allow for free movement of the thighs), as well as the height and width of the top of the desk (table top). The analyzed dimensions of the chairs were seat height and width and backrest width and length. A Cardiomed^®^ anthropometric tape with a precision of 1-mm was used to measure the chairs, and the references of normality followed ABNT NBR 14006 standards.^25^

For data analysis, categorical variables were described as proportions, and continuous variables were reported as means and standard deviations. To identify factors associated with scoliosis, the variables were divided into categories of biological, socioeconomic, lifestyle, and school furniture variables. Prevalence ratios (PRs) and their respective 95% confidence intervals (CIs) were then estimated on a crude and adjusted basis by Poisson regression analysis. We used the hierarchical model with two levels for multivariate analysis, with age, gender, and sexual maturation on the first level of biological and socioeconomic variables and hours of sleep and weight of school supplies on the first level for variables related to lifestyle and school furniture. The variables that had significant differences were then re-analyzed by stepwise Poisson regression analysis to control for confounders. The level of significance was set at 5% in order to minimize type I error.

## RESULTS

The final study sample consisted of 212 school children, among which 41.51% were male (*n* = 88) and 58.49% were female (*n* = 124). The mean age was 11.61 (standard deviation 2.5) years. The prevalence of suspected scoliosis was 58.1% (*n* = 123).

Table 1 presents the sample characteristics. Of the assessed students, 40.1% (*n* = 85) were aged between 10 and 12 years, 58.5% (*n* = 124) were female, 67.5% (*n* = 143) were in puberty, and 62.3% (*n* = 162) were eutrophic. Around 65.6% (*n* = 139) of the assessed students did not touch their fingers to the floor and only 13.2% (*n* = 28) were inactive.

**Table 1.** General characteristics of schoolchildren from the city of Santa Cruz, 2011

Variables	*n*	%
Scoliosis		
Present	123	58
Absent	89	42
Age		
7 to 9 years old	50	23.6
10 to 12 years old	85	40.1
13 to 15 years old	61	28.8
16 to 17 years old	16	7.5
Gender		
Male	88	41.5
Female	124	58.5
Sexual maturation level		
Prepubertal	48	22.6
Pubertal	143	67.5
Post-pubertal	21	9.9
Nutritional status		
Eutrophic	132	62.3
Malnourished	27	12.7
Overweight/obese	53	25
Hand to ground distance		
Fingers touching the ground	73	34.4
Fingers not touching the ground	139	65.6
Tibiotarsal joint angle		
≤90°	16	7.5
>90°	196	92.5
Socioeconomic level		
B_1_, B_2_	28	13.2
C, D, E, F	184	86.8
Level of physical activity		
Active	184	86.8
Low-active	28	13.2
Time watching television every day		
≤2 hours	135	63.7
>2 hours	77	36.3
Habit of sleeping in a hammock		
No	79	81.4
Yes	18	18.6
Hours of sleep every night		
<8 hours	11	5.2
8 to 10 hours	72	34
>10 hours	129	60.8
School supplies weight		
Appropriate	199	93.9
Inappropriate	13	6.2
School supplies mode of transportation		
Appropriate	134	63.2
Inappropriate	78	36.8

Table 2 shows the percentages of children for which the school’s furniture was inappropriate. The chair’s seat height (*n* = 135; 63.7%), the table top’s height (*n* = 157; 74.1%) and width (*n* = 176; 83%) had been the school furniture dimensions with a higher proportion of inadequate measures for the school-aged children.

**Table 2.** Characteristics of school furniture of schools in Santa Cruz, 2011

Furniture dimensions	Appropriate		Inappropriate	
	
	*n*	%	*n*	%
Seat				
Height	77	36.3	135	63.7
Width	162	76.4	50	23.6
Backrest				
Width	200	94.3	12	5.7
Extension	202	95.3	10	4.7
Table top				
Height	55	29.9	157	74.1
Width	36	17	176	83
Seat height to provide free movement of the thighs	184	86.8	28	13.2

The analysis of the association of suspected scoliosis with biological and socioeconomic variables is shown in Table 3. In the crude analysis, scoliosis was positively associated with age between 13 and 15 years (PR 2.94; 95% CI, 1.02–8.45). However, this association disappeared in the adjusted model.

**Table 3.** Model of crude and adjusted association between scoliosis and biological and socioeconomic factors in school children in Santa Cruz, 2011

Variables	Scoliosis		Crude analysis	Adjusted analysis
		
	*n*	%	PR (95% CI)	PR (95% CI)
Age				
7 to 9 years old	21	17.1	1	1
10 to 12 years old	44	35.8	1.48 (0.73–2.99)	1.07 (0.44–2.57)
13 to 15 years old	48	39	5.09 (2.22–11.71)**	2.94 (1.02–8.45)**
16 to 17 years old	10	8.1	2.3 (0.72–7.32)	1.21 (0.28–5.33)
Gender				
Male	40	32.5	1	1
Female	83	67.5	2.43 (1.39–4.26)*	2.03 (1.07–3.85)*
Sexual maturation level				
Prepubertal	17	13.8	1	1
Pubertal	92	74.8	3.29 (1.66–6.52)**	3.70 (1.68–8.17)**
Post-pubertal	14	11.4	3.65 (1.24–10.77)**	3.96 (1.15–13.57)*
Nutritional status				
Eutrophic	72	58.6	1	1
Malnourished	11	8.9	0.57 (0.25–1.33)	0.91 (0.33–2.48)
Overweight/obese	40	32.5	2.56 (1.26–5.23)**	2.5 (1.10–5.67)*
Hand to ground distance				
Fingers touching the ground	31	25.2	1	1
Fingers not touching the ground	92	74.8	2.65 (1.48–4.75)**	2.07 (1.08–4.0)*
Tibiotarsal joint angle				
≤90°	8	6.5	1	1
>90°	115	93.5	1.42 (0.51–3.94)	0.98 (0.3–3.32)
Socioeconomic level				
B_1_, B_2_	17	13.8	1	1
C, D, E, F	106	86.2	0.88 (0.39–1.98)	0.98 (0.39–2.48)

*P < 0.05, **P < 0.01.

CI, confidence interval; PR, prevalence rate.

Girls seem to be more likely to have scoliosis in both the crude model (PR 2.43; 95% CI, 1.39–4.26) and the adjusted model (PR 2.94; 95% CI, 1.02 = 8.45). Pubertal (PR 3.70; 95% CI 1.68–8.17) and post-pubertal (PR 3.96; 95% CI, 1.15–13.57) children were also more likely to have scoliosis. Overweight/obese children (PR 2.5; 95% CI, 1.15–13.57) presented a higher incidence of scoliosis then eutrophic children in the adjusted model. Not being able to touch the ground was also associated with the presence of scoliosis (PR 2.07; 95% CI, 1.08–4.0).

Analysis of the association between suspected scoliosis and behavioral variables with school furniture is presented in Table 4. The habit of sleeping in a hammock was not associated with the occurrence of scoliosis in the crude analysis, but with the adjustment it became negatively associated with the occurrence of scoliosis (PR 0.30; 95% CI, 0.15–0.58). Those who slept more than 10 hours nightly presented less suspected scoliosis (PR 0.07), as did those who slept between 8 and 10 hours nightly (PR 0.09; 95% CI, 0.008–0.91), compared to those who slept less than 8 hours (PR 0.07; 95% CI, 0.007–0.74). Finally, we observed that students whose desks were not tall enough to provide free movement of the thighs were more than three times as likely (PR 3.18; 95% CI, 1.12–9.06) to exhibit gibbosity after adjustment.

**Table 4.** Model of crude and adjusted association between scoliosis and lifestyle and ergonomics in school children in Santa Cruz, 2011

Variables	Scoliosis		Crude analysis	Adjusted analysis
		
	*n*	%	PR (95% CI)	PR (95% CI)
Level of physical activity				
Active	109	88.6	1	1
Sedentary	14	11.4	1.18 (0.80–1.75)	0.90 (0.48–1.71)
Time watching television every day				
≤2 hours	79	64.2	1	1
>2 hours	44	35.8	0.95 (0.54–1.67)	0.95 (0.50–1.8)
Habit of sleeping in a hammock				
No	35	77.8	1	1
Yes	10	22.2	1.57 (0.56–4.40)	0.3 (0.15–0.58)**
Hours of sleep every night				
<8 hours	10	8.1	1	1
8 to 10 hours	43	35	0.15 (0.02–1.22)	0.09 (0.008–0.91)*
>10 hours	70	56.9	0.12 (0.02–0.95)*	0.07 (0.007–0.74)*
School supplies weight				
Appropriate	116	94.3	1	1
Inappropriate	7	5.7	0.83 (0.27–2.57)	0.91 (0.27–3.07)
School supplies mode of transportation				
Appropriate	77	62.6	1	1
Inappropriate	46	37.4	1.06 (0.60–1.88)	0.94 (0.5–1.8)
Height of the chair seat				
Appropriate	52	42.3	1	1
Inappropriate	71	57.7	0.53 (0.30–0.96)*	0.66 (0.33–1.36)
Width of the chair seat				
Appropriate	92	74.8	1	1
Inappropriate	31	25.2	1.24 (0.64–2.38)	1.53 (0.69–3.42)
Width of the chair backrest				
Appropriate	119	96.7	1	1
Inappropriate	4	3.3	0.34 (0.10–1.17)	0.35 (0.71–1.68)
Extension of the chair backrest				
Appropriate	120	97.6	1	1
Inappropriate	3	2.4	0.29 (0.07–1.17)	0.19 (0.03–1.17)
Height of the table top				
Appropriate	35	28.5	1	1
Inappropriate	88	71.5	0.73 (0.39–1.37)	1.42 (0.65–3.11)
Width of the table top				
Appropriate	92	74.8	1	1
Inappropriate	31	25.2	0.74 (0.35–1.56)	0.86 (0.36–2.05)
Seat height to provide free movement of the thighs				
Appropriate	104	84.6	1	1
Inappropriate	19	15.4	1.62 (0.70–3.78)	3.18 (1.12–9.06)*

*P < 0.05, **P < 0.01.

CI, confidence interval; PR, prevalence rate.

Table 5 presents a multivariate analysis of those variables with significant differences in Tables 3 and 4. In the final model, female gender (PR 2.54; 95% CI, 1.33–4.86) and age between 13 and 15 years (PR 5.35; 95% CI, 2.17–13.21) were associated with suspected scoliosis. Sleeping in a hammock was inversely associated with scoliosis (PR 0.44; 95% CI, 0.23–0.81).

**Table 5.** Construction of the final model of association between scoliosis and lifestyle and ergonomics in school children in Santa Cruz, 2011

Variables	Model 1	Model 2	Model 3	Model 4	Final Model
	PR (95% CI)	PR (95% CI)	PR (95% CI)	PR (95% CI)	PR (95% CI)
Gender					
Male	1	1	1	1	1
Female	2.52 (1.27–4.98)*	2.48 (1.26–4.88)**	2.49 (1.28–4.85)**	2.44 (1.26–4.74)**	2.54 (1.33–4.86)**
Age					
7 to 9 years old	1	1		1	1
10 to 12 years old	1.02 (0.4–2.57)	1 (0.41–2.42)	1.03 (0.43–2.47)	1.12 (0.47–2.65)	1.6 (0.75–3.42)
13 to 15 years old	2.67 (0.86–8.24)	2.86 (0.99–8.25)	3.12 (1.11–8.79)*	3.43 (1.23–9.5)*	5.35 (2.17–13.21)**
16 to 17 years old	0.87 (0.18–4.1)	1.09 (0.25–4.72)	1.39 (0.34–5.67)	1.45 (0.36–5.9)	2.32 (0.65–8.26)
Hand to ground distance					
Fingers touching the ground	1	1	1	1	1
Fingers not touching the ground	1.58 (0.78–3.19)	1.64 (0.82–3.28)	1.8 (0.91–3.56)	1.81 (0.92–3.58)	1.78 (0.91–3.49)
Habit of sleeping in a hammock					
No	**1**	**1**	**1**	1	**1**
Yes	0.46 (0.22–0.94)*	0.41 (0.21–0.79)**	0.44 (0.23–0.84)*	0.45 (0.24–0.85)*	0.44 (0.23–0.81)**
Sexual maturation level					
Prepubertal	1	1	1	1	
Pubertal	2.38 (0.99–5.69)	2.24 (0.94–5.3)	2.21 (0.95–5.15)	2.15 (0.92–4.99)	
Post-pubertal	2.82 (0.73–10.81)	2.88 (0.76–10.83)	2.64 (0.72–9.67)	2.54 (0.69–9.24)	
Seat height to provide free movement of the thighs					
Appropriate	1	1	1		
Inappropriate	1.94 (0.72–5.19)	1.81 (0.68–4.81)	1.62 (0.62–4.18)		
Hours of sleep every night					
<8 hours	1	1			
8 to 10 hours	0.16 (0.01–1.58)	0.16 (0.01–1.51)			
>10 hours	0.15 (0.01–1.39)	0.15 (0.01–1.36)			
Nutritional status					
Eutrophic	1				
Malnourished	1.18 (0.41–3.37)				
Overweight/obese	1.87 (0.79–4.45)				

*P < 0.05, **P < 0.01.

CI, confidence interval; PR, prevalence rate.

## DISCUSSION

The results of the present study demonstrate a high prevalence of scoliosis in school-aged children from public schools in the city of Santa Cruz, Brazil. It seems that suspected scoliosis is related to overweight/obese girls aged between 13 and 15 years, during puberty and post-puberty. Regarding lifestyle and ergonomics, we found that sleeping in a hammock and sleeping more than 8 hours a day is negatively associated with suspected scoliosis, whereas inadequate desk height to provide free movement of the thighs is associated with the presence of suspected scoliosis.

The prevalence of suspected scoliosis, assessed using the Adams forward bend test, has varied in the literature. Some studies have shown low prevalence of scoliosis, reporting prevalence of 1.4%^6^ and 2.5%^25^ among school-aged children. Other studies corroborate the high prevalence of scoliosis found in the present study, reporting prevalence of 26%^9^ and 66%^26^; however, these studies did not report an association between high prevalence and the region of the country where the study was conducted.

In this study, the highest prevalence of scoliosis was observed in females, with a positive adjusted association, confirming the results of studies by Lee et al^29^ and Santo et al,^27^ in which the authors reported female-male ratios of 4.5:1 and 1.7:1, respectively, but with no statistical association. On the other hand, Freire^5^ has observed a relatively high prevalence of scoliosis in males (52.9%) compared to our study (41.5%). It is believed that the increased prevalence of scoliosis in women compared to men is justified by the fact that woman tend to grow more than men from ages 11 to 13 years.^30^ These periods of growth spurt contribute to the emergence of postural changes in childhood and adolescence, particularly scoliosis.^8^^,^^30^ The higher proportion of students aged between 13 and 15 years compared to the ages in the sample could have contributed to an increased influence of women in the onset of scoliosis, but this characteristic should be adjusted for in the hierarchical regression model. The adjustment produced by the model neutralizes the possible influence of this disproportion and allows us to infer that females are more likely to have suspected scoliosis.

Another variable positively related to scoliosis in the adjusted model was sexual maturation. Children in Tanner stages 2, 3, and 4 of sexual maturation, who are considered pubertal, are subject to growth spurts and are more susceptible to postural changes.^31^

During growth, body proportions gradually change as the adult body takes form.^32^ According to Santos et al,^1^ growth is greatest during childhood and slows during early adolescence. Growth and sexual maturation greatly influence the development of the curvature of the spine, which may cause an imbalance in the activation of the muscles, leading to scoliosis.^33^ In this study, we observed a high prevalence of shortening of the posterior muscle chain using two methods: the distance from hand to ground and the measurement of the tibiotarsal joint angle. For Santos,^14^ the shortening of the posterior muscle chain suggests that the subjects present a combination of postural pattern disorders, including pelvic retroversion, lumbar adjustment, frontal pelvic balance disorders, and scoliosis.

Regarding nutritional status, this study showed a positive association between overweight/obesity and the presence of scoliosis, in concordance with the findings of Detsch et al^28^ (PR 1.33; 95% CI, 1.19 to 1.48). Brandalize and Leite^34^ indicate that obesity can cause an anterior displacement of the center of gravity, causing adaptations in the vertebral column and lower limbs. Such adaptations may become permanent, causing postural deviations, such as lumbar hyperlordosis with increased compensatory dorsal kyphosis, cervical hyperlordosis, and anteriorization of the head. In the lower limbs, pelvic anteversion can occur, which is associated with internal rotation of hips, valgus knees, and flat foot. Martelli and Traebert^32^ also reported that children with low body weight may also have a higher incidence of postural deviation, which was associated with inadequate school furniture, growth phase of students, and time spent in school.

In this study, we observed an inverse association between scoliosis and sleeping more than 8 hours per night. We hypothesize that prolonged rest allows a greater relaxation of postural muscles, which is achieved when decubitus postures are adopted. Moreover, decreasing the hours of sleep generates an increase in glucocorticoids levels. Increased glucocorticoid levels may induce apoptosis of osteoblasts and osteocytes by activation of caspase-3, which leads to a significant reduction in bone formation and low bone mineral density and may be a possible aggravating factor for scoliosis.^35^

The habit of sleeping in a hammock was investigated in only 97 school-aged children from our sample and presented an inverse association with the presence of scoliosis. Sleeping in hammocks is a habit inherited from the Brazilian Indians; hammocks are a type of bed consisting of a rectangle made of fabric or knitted material and suspended by its ends.^36^ It has been an indispensable and ever-present element in the lives of Brazilians, especially in the poorest areas in the Northeast region, where people use it as a sleeping device.^36^^,^^37^ Hammocks offer the individual an unstable way of resting, which can act as training for the spine muscles when used routinely. However, when used on an occasional basis, it may cause muscle pain consistent with deconditioning to the musculature poorly suited to instability.^38^

Classic inadequate habits related to transporting school supplies and the ergonomics of the schools’ chairs and desks are considered predisposing factors for postural deformities. For example, too high or too low chair seats require the anterior bending of the child’s head and torso for the accomplishment of school tasks, increasing gravitational load on the spine structures.^39^ However, these factors were not associated with suspected scoliosis in this study.

Several limitations of the present study warrant mention. The ergonomic and SSW analysis were recorded on a single day of the week and may not accurately reflect either the school furniture used on a daily basis by the students or the actual weight of the school supplies being transported. A longitudinal study would allow us to more accurately assess the factors related to the development of scoliosis. Another limitation is related to the identification of scoliosis, for which estimated vertebral rotation is essential for its diagnosis (above 10°). Our method of observation may lead to overestimation of the prevalence of scoliosis. Clinically, diagnosis is accomplished through the use of a scoliometer instrument validated for this purpose.^40^ Moreover, our sample size was small when considering the number of assessed variables and the statistical analysis used, despite the fact that the size calculation of the sample had projected a minimum number of 192 schoolchildren and the final sample comprised 212 schoolchildren. The Poisson regression analysis was separated into biological and behavioral factors, which is a limitation of this study because it does not allow assuring the association of the analysis. To minimize this bias, an additional multivariate analysis (Table 5) was performed using only variables that were found to be significant in both models. Only female gender and age between 13 and 15 years old were associated with suspected scoliosis, whereas sleeping in a hammock was negatively associated. A study with a larger sample size should confirm such findings, as these relationships are plausible. Still, other factors, such as race or genetics, that have not been assessed in this study may have influenced the development of scoliosis and should be considered in future studies.

### Conclusion

Although confirmation of our findings will require conducting further studies on the risk factors for the onset of scoliosis, we observed a positive association between the onset of scoliosis and female sex and being 13 to 15 years old, whereas the habit of sleeping in a hammock was negatively associated with the onset of scoliosis. This study revealed the contribution of internal and external exposures to scoliosis prevalence.

## References

[1] Santos CI, Cunha AB, Braga VP, Saad IV, Ribeiro MA, Conti PB, Occurrence of postural deviations in children of a public elementary school in Jaguariúna, Sao Paulo, Brazil. Rev Paul Pediatr. 2009;27:74–80 (in Portuguese).

[2] Souza Júnior JV, Sampaio RM, Aguiar JB, Pinto FJ Profile of postural deviations of the spine in adolescents from public schools in the city of Juazeiro do Norte – CE. Fisioter Pesquisa. 2011;18:311–6 (in Portuguese).

[3] Burwell RG, Dangerfield PH, Moulton A, Grivas TB. Adolescent idiopathic scoliosis (AIS), environment, exposome and epigenetics: a molecular perspective of postnatal normal spinal growth and the etiopathogenesis of AIS with consideration of a network approach and possible implications for medical therapy. Scoliosis. 2011;6(1):26. 10.1186/1748-7161-6-26 22136338 PMC3293085

[4] Graup S, Santos SG, Moro AR Descriptive study of sagittal lumbar spine changes in students of the federal education system in Florianopolis, Brazil. Rev Bras Ortop. 2010;45:453–9 (in Portuguese). 10.1016/S2255-4971(15)30435-3 PMC4799136 27022594

[5] Freire IA, Teixeira TG, Sales CR Postural habits: diagnosis from photographs. Connections. 2008;6:28–41 (in Portuguese).

[6] Rocha JC, Tatmatsu DI, Vilela DA Association between use of school backpacks and scoliosis in adolescents in public and private schools. Motricidade. 2012;8:803–9 (in Portuguese).

[7] Patias P, Grivas TB, Kaspiris A, Aggouris C, Drakoutos E. A review of the trunk surface metrics used as scoliosis and other deformities evaluation indices. Scoliosis. 2010;5:12. 10.1186/1748-7161-5-12 20584340 PMC2906414

[8] Contri DE, Petrucelli A, Perea DC Postural deviation incidence in students of the 2nd to 5th year of elementary school. ConScientiae Saúde. 2009;8(2):219–24 (in Portuguese).

[9] Nery LS, Halpern R, Nery PC, Nehme KP, Stein AT. Prevalence of scoliosis among school students in a town in southern Brazil. São Paulo Med J. 2010;128(2):69–73. 10.1590/S1516-31802010000200005 20676572 PMC10938972

[10] Vasconcelos GA, Fernandes PR, Oliveira DA, Cabral ED, Silva LV Postural assessment of the vertebral column on deaf schoolchildren aged from 7 to 21 years. Fisioter Mov. 2010;23:371–80 (in Portuguese) 10.1590/S0103-51502010000300004

[11] Fornazari LP, Pereira VC Prevalence of scoliosis posture in primary/junior high school pupils. Health Scholl Notebook. 2008;1:1–13 (in Portuguese).

[12] Perez V. The Influence of school furniture and backpack in muscle-skeletal disorders in children and adolescents [Master’s Dissertation]. Florianopolis: Production Engineering College, Federal University of Santa Catarina; 2002 (in Portuguese).

[13] Jenicek M. Epidemiology, evidenced-based medicine, and evidence-based public health. J Epidemiol. 1997;7:187–97. 10.2188/jea.7.187 9465542

[14] Saints. Postural Clinical Diagnosis: A Practical Guide. 5 ed. São Paulo: Summus; 2001 (in Portuguese).

[15] Barros CC, Oliveira MN, Barbosa AR Scoliosis: screening in students from 10 to 15 years old. Rev Saúde Com. 2005;1:134–43 (in Portuguese).

[16] ABEP. Brazilian Association of Research Companies. 2008. Brazilian criteria for economic classification. Available in http://www.abep.org. 2011 (in Portuguese).

[17] Tritschler K. Measurement and evaluation in physical education and sports by Barrow and McGree. 5 ed: Manole, Barueri, SP; 2003 (in Portuguese).

[18] Matsudo VK. Testing in Sports Science. 7 ed. Celafiscs. São Caetano do Sul; 2005 (in Portuguese).

[19] de Onis M, Onyango AW, Borghi E, Siyam A, Nishida C, Siekmann J. Development of a WHO growth reference for school-aged children and adolescents. Bull World Health Organ. 2007;85:660–7. 10.2471/BLT.07.043497 18026621 PMC2636412

[20] Craig CL, Marshall AL, Sjöström M, Bauman AE, Booth ML, Ainsworth BE, . International Physical Activity Questionnaire (IPAQ): 12-country reliability and validity. Med Sci Sports Exerc. 2003;35:1381–95. 10.1249/01.MSS.0000078924.61453.FB 12900694

[21] Guedes DP, Lopes CC, Guedes JE Reproducibility and validity of the International Physical Activity Questionnaire in adolescents. Rev Bras Med Esporte. 2005;11:151–8.

[22] Lipnowski S, LeBlanc CM Canadian Paediatric Society Healthy Active Living and Sports Medicine Committee Abridged version: Physical activity guidelines for children and adolescents. Paediatr Child Health (Oxford). 2012;17(4):209–10. 10.1093/pch/17.4.209 PMC3381667 23543633

[23] Bojikian LP, Massa M, Martin RH, Teixeira CP, Kiss MA, Bohme MT Female pubertal self-assessment of sexual maturation through the use of drawings and photos. Rev Bras Ativ Fís Saúde. 2002;7:24–34 (in Portuguese).

[24] Martin RH, Uezu R, Parra AS, Arena SS, Bojikian LP, Bohme MT Male self-assessment of sexual maturation through the use of drawings and photos. Rev Paul Educ Fís. 2001;15:212–22 (in Portuguese).

[25] ABNT NBR 14006. Brazilian standard. School furniture: chairs and desks to individual student set. 2 ed. 2008 (in Portuguese).

[26] Ugras AA, Yilmaz M, Sungur I, Kaya I, Koyuncu Y, Cetinus ME. Prevalence of scoliosis and cost-effectiveness of screening in schools in turkey. J Back Musculoskelet Rehabil. 2010;23:45–8. 20231789 10.3233/BMR-2010-0247

[27] do Espírito Santo A, Guimarães LV, Galera MF. Prevalence of idiopathic scoliosis and associated variables in schoolchildren of primary public schools in Cuiabá. Rev Bras Epidemiol. 2011;14:347–56 (in Portuguese). 21655700 10.1590/s1415-790x2011000200015

[28] Detsch C, Luz AM, Candotti CT, de Oliveira DS, Lazaron F, Guimarães LK, . Prevalence of postural changes in high school students in a city in southern Brazil. Rev Panam Salud Publica. 2007;21:231–8 (in Portuguese). 10.1590/S1020-49892007000300006 17612467

[29] Lee CF, Fong DY, Cheung KM, Cheng JC, Ng BK, Lam TP, . Referral criteria for school scoliosis screening: Assessment and recommendations based on a large longitudinally followed cohort. Spine. 2010;35(25):E1492–8. 10.1097/BRS.0b013e3181ecf3fe 21102278

[30] Wei-Jun W, Xu S, Zhi-Wei W, Xu-Sheng Q, Zhen L, Yong Q. Abnormal anthropometric measurements and growth pattern in male adolescent idiopathic scoliosis. Eur Spine J. 2012;21:77–83. 10.1007/s00586-011-1960-x 21826498 PMC3252435

[31] Barbosa KB, Franceschini SC, Priore SE Influence of stages of sexual maturation in nutritional status, anthropometry and body composition in adolescents. Rev Bras Saude Mater Infant. 2006;6:375–82 (in Portuguese) 10.1590/S1519-38292006000400003

[32] Martelli RC, Traebert J Descriptive study of spinal postural changes in 10 to 16 year-old schoolchildren. Tangará-SC, 2004. Rev Bras Epidemiol. 2006;9:87–93 (in Portuguese) 10.1590/S1415-790X2006000100011

[33] Tosato JP, Caria PH Assessment of muscle activity on scoliosis. Rev Bras Crescimento Desenvolv Hum. 2009;19:98–102 (in Portuguese).

[34] Brandalize M, Leite N Orthopedic disorders in obese children and adolescents. Fisioter Mov. 2010;23:283–8 (in Portuguese).

[35] Li H, Liang C, Shen C, Li Y, Chen Q. Decreased sleep duration: A risk of progression of degenerative lumbar scoliosis. Med Hypotheses. 2012;78:244–6. 10.1016/j.mehy.2011.10.036 22118954

[36] Freyre G. The Masters and the Slaves. Recife: ed global; 2003 (in Portuguese).

[37] Andrade MC. Hammock. Online School Research, Joaquim Nebuco Foundation [updated Oct 22nd, 2011; cited Dec 08th, 2011]. Available in: http://basilio.fundaj.gov.br/pesquisaescolar/.

[38] Rossi L, Tirapegui J Current concepts about physical exercise, fatigue and nutrition. Rev Paul Educ Fís. 1999;13:65–82 (in Portuguese).

[39] Ainhagne M, Santhiago V Students’ chair and backpack in the development process of bad posture and possible deformities in children aged 8 to 11 years. Colloquium Vitae. 2009;1:01–07 10.5747/cv.2009.v01.n1.v001

[40] Bonagamba GH, Coelho DM, Oliveira AS. Inter and intra-rater reliability of the scoliometer. Rev Bras Fisioter. 2010;14:432–8. 10.1590/S1413-35552010005000025 21049239

